# Grave’s disease induced by radiotherapy for nasopharyngeal carcinoma: A case report and review of the literature

**DOI:** 10.3892/ol.2013.1332

**Published:** 2013-05-08

**Authors:** JIN-AN MA, XUEZHEN LI, WEN ZOU, YAN ZHOU

**Affiliations:** Department of Oncology, The Second Xiangya Hospital of Central South University, Changsha, Hunan 410011, P.R. China

**Keywords:** Grave’s disease, radiotherapy, nasopharyngeal carcinoma

## Abstract

Radiotherapy is an effective treatment for nasopharyngeal carcinoma (NPC). A number of thyroid dysfunctions are induced by damage resulting from the relatively high doses of radiation administered to the thyroid and pituitary gland during radiotherapy. Hypothyroidism constitutes the most frequent type of thyroid dysfunction induced by NPC radiotherapy, while hyperthyroidism, particularly Grave’s disease, is extremely rare. The present study describes the case of a 40-year-old male who presented with Grave’s disease 2 years after receiving radiotherapy for the treatment of NPC. The patient exhibited swelling of the eyes, an increased appetite, decreased levels of thyroid-stimulating hormone, increased levels of triiodothyronine (T3) and thyroxine (T4) demonstrated by the examination of thyroid function and enlargement of the bilateral intraocular rectus revealed by CT scan. The patient’s symptoms were ameliorated following treatment with propylthiouracil and propranolol for 1 month, and the levels of T3 and T4 were restored to normal. The pathophysiological mechanism of radiotherapy-induced hyperthyroidism has yet to be elucidated. Hyperthyroidism is often neglected as several of its clinical manifestations are similar to other complications observed during and following cancer treatment. Therefore, it is necessary to monitor thyroid function following head and neck radiotherapy.

## Introduction

Radiotherapy is an effective treatment for head and neck cancer and also forms the first-line treatment for nasopharyngeal carcinoma (NPC). During NPC radiotherapy, the thyroid may be partially or fully exposed to the radiation field due to excessive cervical lymph drainage. Various thyroid dysfunctions are induced by damage resulting from the relatively high doses of radiation administered to the thyroid during NPC radiotherapy, among which hypothyroidism is the most common. Radiation-induced damage to the pituitary gland may also cause central hypothyroidism. However, hyperthyroidism induced by NPC radiotherapy is extremely rare (1–3). The present study reports a case of Grave’s disease induced by NPC radiotherapy in a patient admitted to The Second Xiangya Hospital of Central South University (Hunan, China). This study was approved by the ethics committee of the SecondXiangya Hospital of Central South University, Changsha, China. Written informed consent was obtained from the patient.

## Case report

### Clinical presentation, diagnosis and treatment

A 40-year-old male was admitted to The Second Xiangya Hospital of Central South University with a neck mass that had been present for 4 months and retractable epistaxis that had been observed for 2 months. The patient had a history of good health with no history of thyroid-related disease. Upon physical examination, several enlarged lymph nodes that were solid and difficult to move, could be palpated in the upper neck. The largest lymph node was located in the upper right side of the neck and measured ∼4.3 cm in size. The largest lymph node in the upper left side of the neck measured ∼3.2 cm. A CT scan of the nasopharynx revealed a mass on the right wall and narrowing of the right parapharyngeal space. An ultrasound of the superficial lymph nodes demonstrated several enlarged lymph nodes in the neck. An examination using a nasopharyngeal fiberscope revealed a mass on the right wall of the nasopharynx and the biopsy results demonstrated a poorly-differentiated squamous cell carcinoma. ECG, plain chest film X-rays and ultrasound examinations of the liver, gall bladder, pancreas, spleen and kidneys all yielded normal results. According to the observed clinical manifestations, the patient was diagnosed with NPC (poorly-differentiated squamous cell carcinoma, T2N2M0, stage III). Concurrent chemoradiation therapy was administered to the patient. Routine radiotherapy at a dose of DT 72 Gy/36 F/7.5 W (6 MV X-ray) was administered to the primary site and a dose of DT 50 Gy/25 F/5 W was administered to the lower neck tangential radiation field (6 MV X-ray). The radiation dose administered to the posterior upper neck was 66 Gy/33 F/7 W (36 Gy/18 F X-ray and 30 Gy/15 F β-ray). Following radiation therapy, residual lymph nodes in the right posterior neck were evident; therefore, a dose of 10 Gy/5 F/10 MeV β-ray was additionally administered. During radiotherapy, two cycles of concurrent chemotherapy, including docetaxel 75 mg/m^2^ IVGTT on day 1 (d1) every 3 weeks and carboplatin 300 mg/m^2^ d1 IVGTT every 3 weeks, were administered. The treatment of the patient was terminated in June 2010. A physical examination subsequent to the treatment demonstrated that the enlarged lymph nodes previously present in the neck were significantly reduced in size. An examination using a nasopharyngeal fiberscope revealed a smooth mucous membrane with no masses.

### Patient follow-up

**Figure 1.**
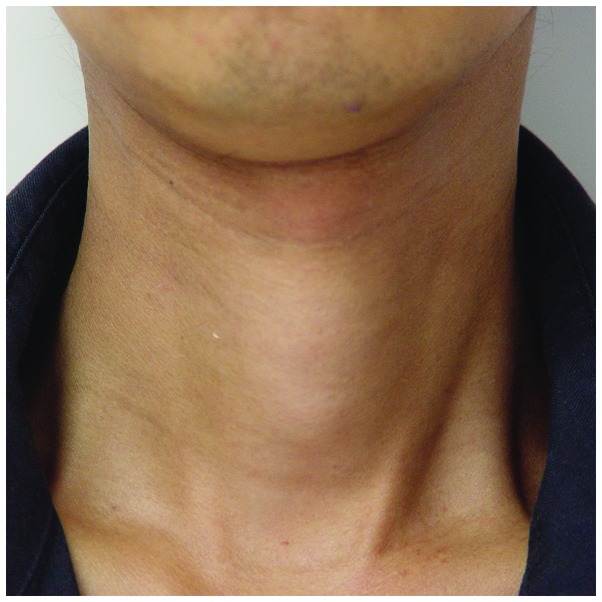
Enlarged and homogeneous thyroid.

**Figure 2.**
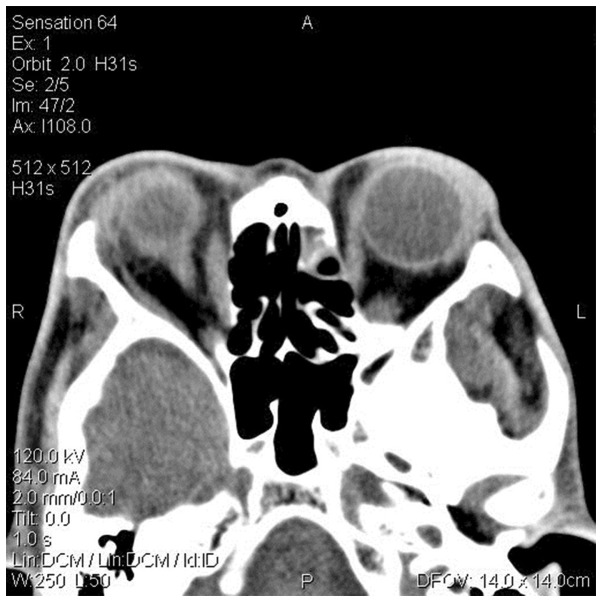
CT scan demonstrating enlargement of the bilateral intraocular rectus.

The patient was regularly followed up at The Second Xiangya Hospital of Central South University, and no local relapse or metastasis was observed. In February 2012, the patient started to complain of photophobia and swelling of the eyes. The symptoms became aggravated in May 2012, and several additional symptoms, including fatigue, insomnia, irritability, palpitations and an increased appetite were noted. A physical examination revealed protrusion of the eyes, with a 16-mm exophthalmos of the left eye and a 17-mm exophthalmus of the right eye. The thyroid was palpated Iº enlargement (Fig. 1). The patient’s heart rate was measured at 102 bpm. The patient felt a tremor in his hands and tongue. No positive signs of rales, tenderness or tension in the lung and abdomen were observed upon examination. An examination of thyroid function revealed the following: Thyroid-stimulating hormone (TSH) <0.005 mIU/l (normal, 0.27–4.20 mIU/l); triiodothyronine (T3), 6.47 nmol/l (normal, 1.30–3.10 nmol/l); thyroxine (T4), 300.5 nmol/l (normal, 66.0–181.0 nmol/l); free (F)T3, 24.90 pmol/l (normal, 3.10–6.80 pmol/l); and FT4, 61.0 pmol/l (normal, 12.0–22.0 pmol/l). An examination of the thyroid-related antibody levels revealed the following results: anti-thyroglobulin (TG), 262.500 IU/l (normal, 0.000–115.000 IU/l); anti-thyroid peroxidase (TPO), 246.300 IU/l (normal, 0.000–34.000 IU/l); anti-thyrotropin receptor (TSHR), 7.930 IU/l (normal, 0.000–1.750 IU/l); and TSH t30<0.011 mIU/l (normal, 2–10 mIU/l). Ultrasonography demonstrated a mild diffusive swelling of the thyroid with abundant blood perfusion. A radionuclide scan of the thyroid showed uniform density with no obvious abnormal uptake or sparse defects. A CT scan revealed rectal thickening (Fig. 2). The patient was diagnosed with Grave’s disease, and propylthiouracil (100 mg orally, three times daily) and propranolol (10 mg orally, twice daily) were consequently administered. One month later, a thyroid function test showed TSH levels of 0.007 mIU/l (normal, 0.27–4.20 mIU/l) and T3, T4, FT3 and FT4 levels within the normal ranges. The patient’s symptoms improved, including those of palpitations, insomnia and fatigue. A physical examination demonstrated a heart rate of 80 bpm. The swelling of the eyes did not improve however, therefore, prednisone was administered.

## Discussion

The thyroid is the largest endocrine organ in the human body. Various thyroid dysfunctions, including hypothyroidism, thyroiditis, benign thyroid tumors and thyroid carcinoma, may be induced by damage caused by head and neck cancer radiotherapy (1). Hypothyroidism is the most commonly observed dysfunction with an incidence of 20–30% (1). However, hyperthyroidism induced by radiotherapy is extremely rare and the earliest studies reporting this condition date back to the 1970s (2,3). In the present study, the patient developed hyperthyroidism and/or Grave’s disease following external radiation therapy for a non-thyroid carcinoma (2,3). Grave’s disease is the most common cause of hyperthyroidism, constituting 80–85% of all cases. The incidence of radiotherapy-induced Grave’s disease is 0.1–0.5% (4). It has been reported that the incidence of Grave’s disease in the population that has received thyroid radiation is 7–20-times higher than for those who have not received thyroid radiation (45 neck carcinoma may lead to thyroid damage. The majority of patients present with clinical or subclinical hypothyroidism, while a limited number present with thyrotoxic symptoms, including hyperactivation of the neural, circulatory and digestive systems and hypermetabolism (1). Thyrotoxicity may be divided into two subtypes, hyperthyroidism and non-hyperthyroidism, according to the function of the thyroid. It has been reported that non-hyperthyroid thyrotoxicosis (transient thyroiditis or unsymptomatic thyroiditis) may be induced following head and neck radiotherapy for thorax cancer (6), Hodgkin’s lymphoma (7–9) and tonsil carcinoma (10). Slacmeulder *et al* (11) reported the cases of 2 children who presented with hyperthyroidism following head and neck radiotherapy; three years after the radiotherapy, one of the patients presented with medulloblastoma, hyperthyroidism, diffusive thyroid enlargement and negative activity of the anti-TSH receptor antibody. Methimazole was effective in treating the symptoms. All the observed clinical manifestations met with the diagnostic criteria of Grave’s disease.

To the best of our knowledge, the present study is the first in China to report that Grave’s disease may be induced by radiotherapy of the head and neck. In the present patient, hyperthyroidism, diffusive thyroid enlargement, protrusion of the eyes and positive anti-thyroid antibody activity, appeared 2 years after the radiotherapy for NPC. Taken together, these symptoms led to the diagnosis of Grave’s disease. The pathophysiological mechanism of radiotherapy-induced hyperthyroidism is unclear. One potential mechanism is that the release of antigen subsequent to radial thyroid damage facilitates the production of thyroid-related antibodies, which leads to chronic autoimmune thyroiditis (12). Another potential mechanism is considered to be the direct cytotoxic activity of the radiation (13). The precise mechanism of radiation-induced hyperthyroidism requires further investigation.

Hyperthyroidism is a rare complication of head and neck cancer, which may be neglected as a result of exhibiting similar clinical manifestations to other complications during and following the treatment of cancer. In addition, other thyroid dysfunctions, including hypothyroidism, are commonly observed following head and neck radiotherapy. It is therefore necessary to monitor thyroid function following head and neck radiotherapy.
